# Indiana Veterinary College

**Published:** 1897-05

**Authors:** 


### INDIANA VETERINARY COLLEGE.

The annual commencement exercises were held March 26th at the college building, 18–24 S. East Street. The exercises consisted of addresses by Dr. L. A. Greiner, Secretary of the College, Dr. Thomas Gaddes, Dr. E. H. Pritchard, and Dr. F. A. Mueller, after which Dr. T. L. Armstrong awarded the diplomas to the following: Clarkson Gause, Carthage, Ind.; David Waugh, Pittsburg, Pa.; Robert F. Harper, Indianapolis, Ind. After the exercises the graduates of the Indiana Veterinary College met and an Alumni Association was organized. The following officers were elected: Thomas Gaddes, M.D., V.S., President; Joseph Creedon, V.S., Vice-President; L. A. Greiner, V.S., Secretary; F. A. Mueller, Ph.G., V.S., Treasurer. The following were elected trustees to serve one and two years respectively: Dr. Harry Smock, Franklin, Ind.; Dr. Wilbur Ramsey, Middletown, Ind.; Dr. J. B. Heaton, Bloomfield, Ind.; Dr. S. Rodebaugh, New Augusta, Ind.; Dr. Clark Gause, Carthage, Ind. The meeting then adjourned, and a general good time was had the balance of the afternoon.

